# A Holarctic Biogeographical Analysis of the Collembola (Arthropoda, Hexapoda) Unravels Recent Post-Glacial Colonization Patterns

**DOI:** 10.3390/insects2030273

**Published:** 2011-06-29

**Authors:** María Luisa Ávila-Jiménez, Stephen James Coulson

**Affiliations:** 1 Department Arctic Biology, University Centre in Svalbard, P.O. box 156. 9171, Longyearbyen, Norway; 2 Ecological and Environmental Change Research Group (EECRG), Department of Biology, University of Bergen, p.b. 7800, N-5200 Bergen, Norway; 3 Department Arctic Biology, University Centre in Svalbard, P.O. box 156. 9171, Longyearbyen, Norway; E-Mail: steve.coulson@unis.no

**Keywords:** dispersal, colonization, distribution range, glacial refugia, Gaussian mixture clustering, Arctic

## Abstract

We aimed to describe the main Arctic biogeographical patterns of the Collembola, and analyze historical factors and current climatic regimes determining Arctic collembolan species distribution. Furthermore, we aimed to identify possible dispersal routes, colonization sources and glacial refugia for Arctic collembola. We implemented a Gaussian Mixture Clustering method on species distribution ranges and applied a distance- based parametric bootstrap test on presence-absence collembolan species distribution data. Additionally, multivariate analysis was performed considering species distributions, biodiversity, cluster distribution and environmental factors (temperature and precipitation). No clear relation was found between current climatic regimes and species distribution in the Arctic. Gaussian Mixture Clustering found common elements within Siberian areas, Atlantic areas, the Canadian Arctic, a mid-Siberian cluster and specific Beringian elements, following the same pattern previously described, using a variety of molecular methods, for Arctic plants. Species distribution hence indicate the influence of recent glacial history, as LGM glacial refugia (mid-Siberia, and Beringia) and major dispersal routes to high Arctic island groups can be identified. Endemic species are found in the high Arctic, but no specific biogeographical pattern can be clearly identified as a sign of high Arctic glacial refugia. Ocean currents patterns are suggested as being an important factor shaping the distribution of Arctic Collembola, which is consistent with Antarctic studies in collembolan biogeography. The clear relations between cluster distribution and geographical areas considering their recent glacial history, lack of relationship of species distribution with current climatic regimes, and consistency with previously described Arctic patterns in a series of organisms inferred using a variety of methods, suggest that historical phenomena shaping contemporary collembolan distribution can be inferred through biogeographical analysis.

## 1. Introduction

The historical frame in which contemporary biogeographical patterns in the Arctic have been shaped is as yet a mystery for most species of invertebrates. Furthermore, for most species, whether any overall pattern defines their Arctic distribution has never been clarified, although it is clear that not all species are found everywhere. Environment and physiological limitations, together with dispersal abilities, are frequently highlighted as major factors determining the ranges of species in different taxa [1], but more recently it has been highlighted that historical influences can contribute to contemporary patterns of biodiversity to a similar or greater extent than contemporary climatic regimes [2]. The current geographical distribution of species reflects not only the ability of given species to survive the environmental conditions, compete and successfully reproduce in a particular location, but also their ability to have successfully colonized the area once the appropriate niche became available. The Arctic thus arises as an ideal platform for studying the large scale dispersal abilities of terrestrial invertebrates as, for much of the area, a relatively accurate date estimate can be given to the “*opening of the niche*” event.

Ice ages have been repeatedly shown to have a measurable impact on current Arctic diversity patterns through species range reduction (bottlenecks) and expansion episodes [3–8]. Most of the high Arctic was covered by permanent ice during the Wisconsinan/Weischelian glacial episode, although the exact area and timing of the Last Glacial Maximum (LGM) extension of ice varied between regions [9]. Outlet glaciers of the Laurentide ice sheet in the Canadian Arctic retreated rapidly approximately 12- 10 Ka B.P. [10], while late glacial nunataks showing pioneer dwarf- shrub vegetation were present in southern central Scandinavia 16 Ka B.P. [11]. Recent analysis however points at a Last Scandinavian Ice Sheet deglaciation mostly due to surface thinning rather than marginal retreat [12]. Areas such as Beringia and most of Siberia as far as the Taymyr peninsula were ice-free for most if not all of the glacial period [13–15], leaving a broad area in the eastern Palaearctic that could have potentially provided glacial refugia. The Kara Ice Sheet could have left ice-free ground available along its western sector as early as 15 Ka B.P. [14]. Knowledge of patterns of the LGM ice sheet extension and thickness are in general based on large scale reconstructions, often with doubtful boundaries and low resolving power in the context of identifying small islands of ice-free ground (see [14,10,11]). This has led to the concept of cryptic refugia [16], which are areas, probably scattered and in low densities, where organisms could have persisted during major glacial episodes. In the instance of the Svalbard archipelago, it has been suggested that some ice-free areas could have remained in Murchinsonfjorden [17–19] and Danskøya [20], but the currently available reconstructions cannot accurately assess whether those areas were ice-free or covered by a thin cold-based ice sheet [17,19]. In most of the possible ice-free areas in the high Arctic, the environment is thought to have been harsh enough that no soil mesofauna could have survived *in situ*. Genetic data suggest that no Arctic plants were present in high Arctic regions prior to the abrupt end of the LGM 10 Ka. B.P. [5], when large periglacial areas opened for colonization. Several authors nonetheless, point to survival of a number of invertebrate groups in the arguably more severe conditions of continental Antarctica [21–23], thereby challenging current reconstructions of the glacial history of the Antarctic continent [21]. Furthermore, different collembolan ecotypes show different adaptations to harsh environments, and even cryophilic glacial dweller collembolan species have been described [24,25]. This challenges the often unquestioned concept that *nothing could have survived in the high Arctic*, and replaces it with the question of *what could have survived and where?*

How past colonization patterns shaped the current spatial distribution of species is of special interest in determining the historical forces driving species biogeography. For many species, the chance of winning the battle against global extinction depends on their ability to both live in a range of environments and the ability to track them [26]. Even though this is commonly assumed, few studies have applied large-scale biogeographical analyses as a tool to evaluate present biodiversity in the Arctic, even though macroecological approaches are increasingly applied elsewhere [1,2,27–31]. In the case of Arctic soil invertebrates, diversity and distribution patterns are often poorly described or understood [32].

Soil invertebrates play an important role in ecosystem functioning, for example having an impact on nitrogen fixation [33] and carbon availability [34], modifying soil structure and properties [35], indirectly enhancing seedbank persistence [36] or acting as fungal spore vectors [37]. Decomposer communities are essential for ecosystem function, also accounting for the major energy and nutrient pathways in polar terrestrial ecosystems, and the soil microarthropod community fulfils an important role accelerating microbial and fungal uptake, immobilization and mineralization of nutrients required by the autotrophic community [38]. Large scale alterations in distribution patterns of soil invertebrates could thus have significant effects on ecosystem change and development over time.

In most cases, the distribution records of Arctic species are confusing or incomplete, thus data on aspects such as biogeography, dispersal, expansion or reduction of species ranges is difficult, often impossible, to extract. Collembola are an abundant and widespread element of the soil fauna about which there is considerable information available for the polar regions, from molecular biology to ecophysiology [22,39–41], making it an ideal group for macroecological analysis. Early studies showed how collembolan distribution can be determined both by broad zoogeographical factors and short-range ecological determinants. At a global level evidence for a pre- Gondwanaland origin for the Collembola can be inferred through group biogeography [42]. More recent studies suggest present day patterns in collembolan distribution in Antarctica to be the result of the interaction between historical and environmental drivers [43]. In the case of Arctic springtails, the taxonomy has recently been subject to comprehensive revision, and diversity and distribution are well described regionally [32,44–49].

Recently, efforts have been made to standardize the existing knowledge into a homogeneous dataset, not only at the regional level [32,50], but for the entire Arctic area [44]. Nonetheless, these data are yet to be used in a comprehensive biogeographical analysis. Arctic springtails possess several attributes which make them particularly suitable to large-scale biogeographical analysis, for example, many species are highly flexible in regard to dietary requirements, and possessing long and flexible life cycles that permit advantage to be taken of short periods of favorable conditions [51–53].

In a rapidly changing Arctic, with temperatures expected to rise up to 5 °C by the end of 21st Century [54], current biogeographic patterns may well soon belong to the past. Our study focuses on deciphering historical patterns underlining the existing biogeography in Arctic areas and determining whether, in areas of recent colonization, historical influences on these distribution patterns are more strongly visible than ecophysiological constraints. Distance-based test statistics have been developed to test for the existence of clusters of species according to their ranges [55]. By implementing a multivariate statistical approach on species presence/absence data, in combination with ten years' (1996–2005) environmental data from 15 Arctic areas as indicative of contemporary climatic regimes, we aimed to address the following questions concerning the Collembola: (1) Does any biogeographical pattern exist in the Arctic? (2) Can this pattern be explained solely by contemporary climatic regimes? (3) Do biogeographical patterns in the Arctic relate to historical geographical events? We additionally attempt to provide evidence or interpretation as to (4) Could any terrestrial biota have survived in the Arctic in situ throughout the LGM?

## 2. Experimental Section

### 1.1. Diversity Data Collection

**Figure 1:**
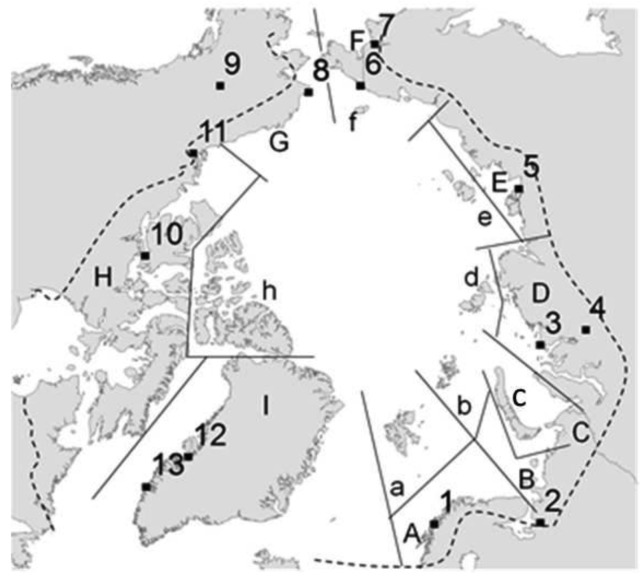
Area nomenclature and location as defined by Babenko and Fjellberg [44], including indication of weather stations used in the study indicating type of data obtained as t: temperature and p: precipitation (1: Tromsø (t&p), 2: Arkhangelsk(t&p), 3: Ostrov Dikson, (t), 4: Dudinka (p), 5: Tiski (t&p), 6: Shmidta (t), 7: Anadyr' (p), 8: Cape Lisburne (t), 9: Fairbanks (p), 10: Cambridge Bay (t), 11: Inuvik (p), 12: Illulisat (t), 13: Nuuk (p)). Capital letters define continental areas and lower case indicate Arctic islands (A: western Europe, B: eastern Europe, C: Urals, D: west and mid-Siberia, E: east Siberia, F: North- East Asia, G: western America, H: eastern America, I: Greenland, a: Svalbard, b: Franz Josef Land, c: Novaya Zemlya, d: Severnaya Zemlya, e: New Siberian Islands, f: Wrangel island, h: Queen Elisabeth Islands and Ellesmere island).

A presence/absence (1/0) matrix was created from the data compiled at the Arctic collembolan catalogue by Babenko and Fjellberg [44], considering 16 geographical areas also as defined by Babenko and Fjellberg [44] (Figure 1), Area C (Urals), although indicated in the main description table (Table 1), was excluded from the statistical analyses as this region has been considerably under-sampled in comparison with the remaining areas and the diversity index is unrealistically low [56]. Additionally, and to avoid bias in the dataset, species with dubious records (marked as a ‘?’ in Babenko and Fjellberg [44]), likely recent introductions into Arctic regions (for example *Orchesella*), recently described species (species described from 2005 onwards, as they may have been overlooked in other regions) and species with unresolved status were not included in the current study. Thus 358 species were considered in the study from a total of 390 species described from Arctic areas.

### 1.2. Environmental Data

Information on contemporary climatic regimes from each mainland area was gathered, including factors previously identified as affecting collembolan species distribution: temperature [39,57] and precipitation [58]. Available meteorological data from the Arctic island archipelagos is in most cases scattered and often biased towards summer measurements. Therefore environmental data from the Arctic islands were not included.

#### 1.2.1. Temperature

Temperature data for the period 1996–2005 were retrieved from the US National Oceanic and Atmospheric Administration (NOAA) [59], available through the World Meteorological Organization (WMO) [60]. A representative weather station was selected for each Arctic area (A: Tromsø airport; B: Arkhangelsk; D: Ostrov Dikson; E: Tiksi; F: Shmidta; G: Cape Lisburne; H: Cambridge Bay; I: Illulisat), All temperature stations are within an altitude range of 0 to 40 m.a.s.l.

#### 1.2.2. Precipitation

Precipitation data were obtained as yearly averages from the WMO data centre [60], where possible sourced from the same weather stations as the temperature data (A: Tromsø airport; B: Arahngelsk; D: Dudinka; E: Tiksi; F: Anadyr'; G: Fairbanks; H: Inivuk; I: Nuuk.) All precipitation stations are located in an altitude range from 0 to 80 m.a.s.l. with the exceptions of Dudinka (200 m.a.s.l.) and Fairbanks (140 m.a.s.l.).

### 1.3. Statistical Analyses

#### 1.3.1. Gaussian Mixture Clustering

Cluster analysis was carried out using R-Package MCLUST [61–63] to perform a distance-based parametric bootstrap test for clustering. This will reveal background biogeographical patterns based on presence-absence data (clustering of species ranges). MCLUST uses Kulczynski distance measures, which approximates to 1 when ranges do not overlap, 0 for complete overlap, and to 0.5 when one range is a subset of the other. The procedure provides a number of meaningful clusters (K), defining each cluster as a series of biotic factors (species) [64]; MCLUST requires previous identification of those points that do not fit in any cluster. These are considered as noise by the software NNclean included in the package [65,66]. This analysis assumes two main characteristics for taxon ranges: (1) the occurrence of a taxon in one geographical unit increases the probability of occurrence in neighboring units and (2) different geographical units vary in their potential to contain species diversity [55]. MCLUST thus computes a Multidimensional Scaling (MDS) based on Kulczynski distances and applies maximum likelihood Gaussian mixture clustering with noise to the MDS points, considering normal components as stable patterns in the data [67]. A parametric bootstrap test was performed on the Kulczynski distance matrix using statistics test distratio with 1000 bootstrap simulations. Clusters were plotted on each geographical location using ESRI ArcMap.

#### 1.3.2. Polynomial Models and Analysis of Variance

Polynomial regression was performed, as it does not assumes *a priori* linearity in the response, between biodiversity and cluster distribution and the environmental variables using SigmaPlot (V. 11, Systat Software Inc.). Analysis of variance in ranks was applied to the presence/absence data among areas, and weather data among areas, using the same software, to establish pairwise significant differences between areas due to temperature and diversity data.

## 3. Results

### 3.1. Clustering

Gaussian mixture clustering was significant for K = 9 (statistics value for original data = 0.26; mean for null data = 0.335; *p* < 0.05). From the 358 species included in the study, 169 were considered noise by NNclean, while the remaining 189 were allocated to any of the 9 clusters. Cluster 1 was composed mainly by Palaearctic/Atlantic species, clusters 2, 5 and 7 by mainly Nearctic species, clusters 3, 4, 6 and 8 mainly by Palaearctic species and cluster 9 by Holarctic species (Table 2).

**Figure 2:**
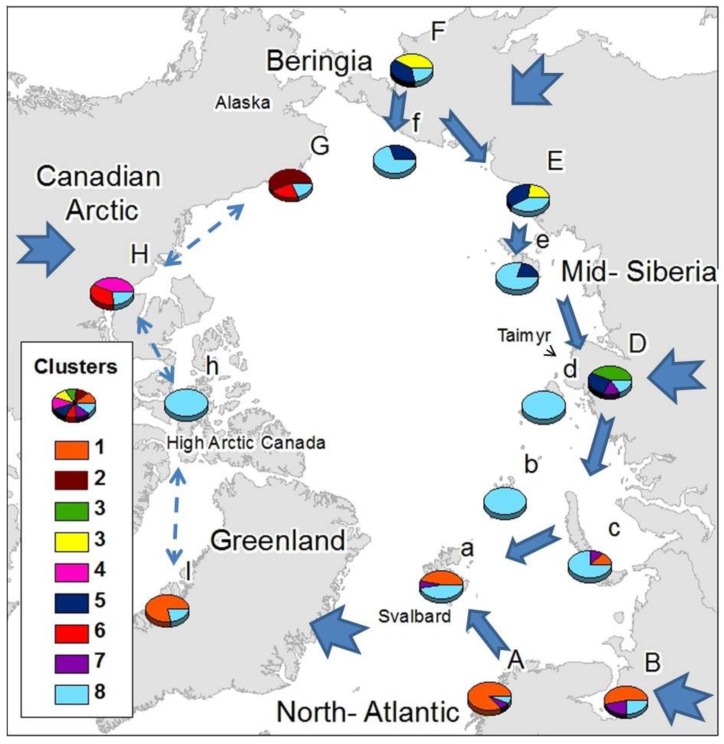
Gaussian mixture clustering geographically represented as number of species allocated to each cluster at each Arctic area. Arrows represent suggested dispersal routes to (thick arrows) and within (thin arrows) Arctic areas. Dashed arrows indicate areas of unresolved main dispersal routes.

Common elements were present within Siberian areas, Atlantic areas and the Canadian Arctic (Figure 2), with some wide-ranging species shared among all them. Just one cluster (cluster 9) was found throughout the Arctic, comprising exclusively cosmopolitan and holarctic, species: *Agrenia bidenticulata* (Tullberg, 1876), *Folsomia bisetosa* Gisin, 1953, *Folsomia quadrioculata* (Tullberg, 1871), *Pseudoisotoma sensibilis* (Tullberg, 1876), *Megalothorax minimus* (Willem 1900), *Oligaphorura groenlandica* (Tullberg, 1876) and *Sminthurides malmgreni* (Tullberg, 1876). This is in contrast to the Beringian area (F, G) which showed a high number of species characteristic from that area (22 species from cluster 2 exclusive from G, and the 13 species from cluster 4, which are mainly found in F), although some species characteristic of area F also appear in area E: *Tetracanthella martynovae* Potapov, 1997, *Psyllaphorura sensillifera* (Martynova, 1981), *Protaphorura neriensis* (Martynova, 1976), *Anurida bondarenkoae* Tshelnokov, 1988) (Table 2).

Three clusters appear uniquely in one mainland area and are absent from their respective Arctic island groups: Cluster 2 in the American side of Beringia, comprising species present in Western America (G); Cluster 3 in mid-Siberia including species present in west and mid-Siberia (D); and Cluster 5 in the Canadian Arctic. The American-Beringian cluster (Cluster 2) appears as unique and different to the main Asian-Beringian cluster (Cluster 4) (Figure 2). Most of the species that colonize Atlantic areas have European-Western Palaearctic distributions, while species colonizing Eastern Siberia have mostly Asiatic distributions and species found in the Canadian Arctic are mainly Nearctic (Table 2). Species distributed throughout the Arctic are mostly not exclusively known from Arctic areas, while limited-range or endemic species appear in Beringia, mid-Siberia, the Canadian Arctic and Greenland (Table 3).

High Arctic islands seem to be colonized primarily by wide-ranging species, although that is not the case for the Svalbard archipelago which comprises elements from the common holarctic cluster (9) and the Atlantic and East European clusters. There is just one species currently thought to be specific to the high Arctic islands not present elsewhere in the Arctic, *Folsomia altamontana* Yosii, 1971, described from Severnaya Zemlya and New Siberian Islands.

### 3.2. Polynomial Models and Analysis of Variance

Geographical areas were found to be clearly different whether considering species composition (analysis of variance on ranks, H = 84.80, df = 7, *p* < 0.05) or daily temperature (period 1996–2005) (analysis of variance on ranks, H = 5091.93, df = 7, *p* < 0.05). The *post-hoc* Dunn's test for pairwise differences between areas revealed that pairwise differences between areas due to temperature were not the same as the pairwise differences between areas due to species distribution (Table 4).

**Figure 3:**
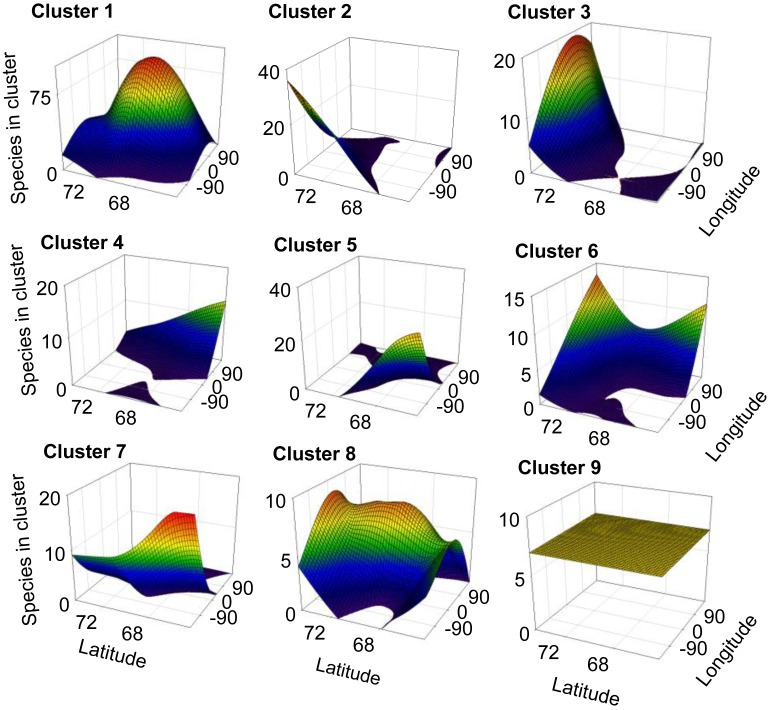
Main distribution of the species in a longitude and latitude axis system for each species cluster, as number of species of the cluster present at each longitude/latitude coordinate.

**Figure 4:**
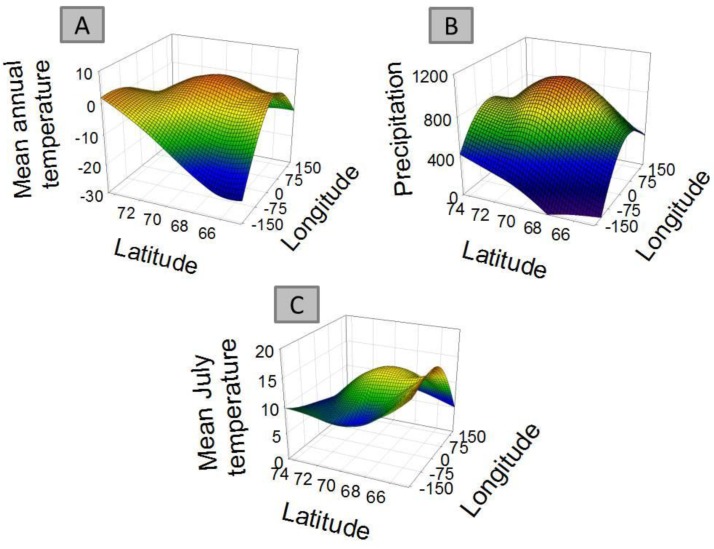
Main distribution of average temperature (**A**), precipitation (**B**), and mean temperature of the warmest month (July) (**C**) data in longitude and latitude axis system, as average temperature for the period 1996– 2005 (A) and average yearly precipitation (B) recorded at each longitude/ latitude coordinate.

For 8 out of the 9 clusters found by Gaussian mixture clustering, no clear relation was found between the geographical distribution of the species present in each cluster and the environmental variables accounted. Species belonging to each different cluster shows a geographical distribution (Figure 3) which does not relate to the curved representing average temperature (Figure 4A), temperature of the warmest month (July) (Figure 4B), or precipitation (Figure 4B). The distribution of cluster 1 shows a linear relation with both precipitation (r^2^ = 0.830, F = 29.255, *p* < 0.005) and mean annual temperature (r^2^ = 0.593, F = 8.725, *p* < 0.05).The distribution of cluster 6 shows a second grade relation with mean temperature of the warmest month (r^2^ = 0.59, F = 11.40, *p* < 0.05). The distribution of species considered as noise by Gaussian Mixture Clustering does not show a relationship neither with mean annual temperature (r^2^ = 0.210, F = 1.591, *p* >0.05), nor with precipitation (r^2^ = 0.172, F = 1.244, *p* > 0.05)

## 4. Discussion

If the biogeography of Arctic Collembola is determined by current climatic regimes, distribution patterns would follow climatic features rather than defined geographical features. The pattern identified by our analyses, however, appears to be more strongly determined by recent historical events such LGM ice extent and glacial refugia, and colonization patterns.

### 4.1. Biogeographical Pattern for Arctic Collembola

The geographical distribution of clusters (Figure 2) showed the existence of a relationship within different Atlantic areas (A, a, I, as far as area B in East Europe), western Siberia (B, c, D), eastern Siberia (E, F, D), Asian-Beringia (E,F), Alaskan-Beringia (G) and the Canadian Arctic (H). Greenland appears as more closely related to the European Arctic than to the Canadian Arctic. The western and eastern Palaearctic showed a different cluster structure, sharing just the common Holarctic element. Specific clusters, exclusive to a particular region were found in mid-Siberia, Asian-Beringia, Alaskan-Beringia and the Canadian Arctic, while the high Arctic islands possessed mainly components from the most widespread cluster composed of wide-ranging species. Only a small number of cosmopolitan species were present throughout the Arctic (Table 2).

Species colonizing each defined Arctic area also tended to share specific regional distributions: species colonizing the Canadian Arctic were mainly Nearctic, those colonizing Scandinavia had mainly a western Palaearctic distribution and species colonizing the eastern Palaearctic had mostly Asiatic-Beringian distributions. Endemic species were present in mid- and western Siberia (D), particularly in the Taimyr Peninsula, in Beringia (F and G) and only a single endemic species occurred in eastern North America (H) and Greenland (I) (Table 3).

### 4.2. Effect of Contemporary Climatic Regimes on Arctic Collembolan Distribution

Factors as precipitation and temperature have been previously indicated as limiting the distribution of collembolan [39,57,58]. Differences found in climate between areas also do not match closely those found in species distribution (Table 4). Clustering do not follow environmental parameters either—for instance areas more distantly related in terms of climatic regimes (e.g., Scandinavia (A) and mid-Siberia (D)) share a cluster which is not share by Greenland (I), which has an intermediate temperature and precipitation regime. Each cluster shows a defined distribution, often sharply skewed towards certain geographic locations (Figure 3), showing no consistency with temperature and precipitation gradients (Figure 4A and 4B). Additionally, species considered as noise do not show a relationship with environmental parameters. Although contemporary climatic regimes may play a role in species distribution, this is not capable of explaining the geographical variation found. Additionally, adjacent areas are more likely to be influenced by similar climatic regimes, for instance the western Palaearctic, Svalbard Archipelago and Greenland are under the influence of the North Atlantic Oscillation [68]. Therefore, there is a possibility that these environmental parameters are in effect autocorrelated with the distribution clusters.

### 4.3. Relationship between Biogeographical Pattern and Recent Historical Events

Northbound colonization patterns as the Arctic deglaciated would explain why most species colonizing Atlantic areas have European-western Palaearctic distributions, species colonizing eastern Siberia have mostly Asiatic distributions and species found in the Canadian Arctic are mainly Nearctic (Table 1). The strong Atlantic influence on distribution clusters of the Greenland area disagrees with previous Holarctic studies both in Tardigrada [69] and in Cladocera [70] where a Nearctic origin of the Greenland fauna was suggested. Nevertheless, Cladocera and Tardigrada, aquatic groups presenting drought resistance stages, have been highlighted as good airborne dispersers [70]. On the other hand for the non-anhydrobiotic springtails, long distance aerial dispersal in polar areas is unlikely [71] due to mortality following rapid desiccation [72], In spite of collembolan being considered truly soil dwellers, some surface active species can show higher dispersal potential and resistance to desiccation, and aerial dispersal should not to be underestimated for short distance dispersal in moist conditions. Springtails have, however, been shown to survive long periods on sea- water [73], and ocean currents, together with glacial refugia, may have an important role in delineating Antarctic distributions [74]. Collembola has been described to been passively disperse by water [75,76]. Should species of Collembola be effective sea water dispersers, it raises the question of why there is no obvious connection between the Palaearctic and Nearctic areas even though ocean currents and ice flow patterns show a level of physical connection between the Palaearctic and Nearctic across the Arctic Ocean [77] and given that springtails have also been shown to survive extended periods below zero [78–80]. Plant molecular studies have also failed to support the traditional hypothesis that considers the north Atlantic an important dispersal barrier between Palaearctic and Nearctic areas [7,81,82]. Abbott *et al* [83], applying RFLP techniques in chloroplast DNA of the Arctic plant species *Saxifraga oppositifolia* L., obtained a general Arctic structure showing a series of similarities with the pattern obtained in the current study (Figure 2). A Beringian component was shared among eastern Siberia areas as far as Taymyr, a mid-Siberian component was shared as far as Scandinavia and the Svalbard archipelago and an Atlantic component was present from Scandinavia to western Greenland. These patterns were associated with glacial refugia and post-glacial colonization phenomena. In both cases, either gaussian mixture clustering in the distribution of the Arctic springtails or cp DNA haplotype distribution of *S. oppositifolia*, the eastern boundaries of the Atlantic component occur west of the Taimyr Peninsula, while the Taimyr forms the western boundary of the Beringian cluster. The link between Atlantic and west and mid-Siberia shown by cluster 8 suggest a link between these areas and could be indicative of post-glacial colonization from LGM ice-free areas in mid-Siberia.

Beringia refugia as determining factor on biogeographical structure of terrestrial fauna and flora have been repeatedly emphasized [4,5,84]. Insights into the colonization of Siberia from Beringia, as described for Arctic plants [83], are provided from the extension of cluster 6 from Beringia (F) to mid-Siberia (D) as well as cluster 4 into eastern Siberia (E). The low number of species colonizing the high Arctic islands could suggest recent colonization. Nonetheless, the high Arctic islands, although colonized by a lower number of species, appear to have a higher species diversity than that which would be expected by island biogeography theory [85]. In the case of high Arctic Canada and many Siberian Arctic islands, only wide-ranging species colonize them rather than species commonly found on the closer mainland (Figure 2). This suggests that mostly wide-ranging and competent dispersers have had the ability to reach these areas. Wrangel island and the New Siberian islands could be also colonized from eastern Siberia and Beringia, their closest mainland with which they share cluster 6 as has been previously suggested based on local fauna descriptions [50], Novaya Zemlya could have been colonized from either or both Eastern Europe and Mid Siberia, while the Svalbard archipelago seems to have been colonized both from distant (mid-Siberia) and close (Atlantic), mainland sources.

The disjunct distribution of the high Arctic species *Folsomia altamontana* Yosii, 1971, which is present not only in Severnaya Zemlya and the New Siberian islands but also the Himalaya and Putorana Plateau, could suggest either a magnificent ability for long distance dispersal, relict populations for what during glaciation was a more widespread species, or a cryptic speciation event, as has recently been suggested for the Antarctic *Friesea grisea* (Shaffer 1891) [41]. *Folsomia altamontana* is thus an interesting target for further phylogeographical analysis.

### 4.4. Could Collembola Have Survived in Situ in the Arctic during LGM?

Endemic collembolan species (narrow range endemism) occur in north and mid-Siberia (D), Beringia (F and G), the Canadian Arctic (H) and Greenland (I), all of which have been previously indicated as including possible refugia during LGM by a number of authors and for a series of taxa [81,82,86–88]. The two possible glacial refugia that could be identified in mid-Siberia, east of Ural Mountains (D), and Beringia (F and G), defined by cluster characteristics (Figure 2) and inferred from the raw data through presence of endemic species (Table 3), agree with glacial refugia described for Arctic plants [82,6]. In contrast, the exclusive cluster in the Canadian Arctic seems to be composed mainly by species widespread (5) in North America, suggesting a main dispersal route to the Arctic rather than a possible refugium, as discussed in the case of Arctic Tardigrada [69]. Additionally, no indication of glacial survival in the high Arctic islands can be found in the current analysis of Arctic Collembola, although occurrence of endemism in other invertebrate groups could indicate otherwise [89]. A particularly interesting case is provided by the high Arctic islands in the Canadian Archipelago, including Ellesmere island and Baffin island. This region was suggested by Hultén [81] as a possible glacial refugium as it remained largely unglaciated throughout LGM and was postulated as a source for post-glacial Arctic recolonization for plant species such as *Dryas integrifolia* [88] or the collared lemming *Dicrostonyx groenlandicus* [86]. However, this area does not show other patterns which might be expected in an area of recent colonization, for example no endemic or limited range species, rather being colonized by a low number of species, and possessing mostly cosmopolitan or Holarctic species. Species-level biogeographical analysis however overlooks obscure regional endemism due to cryptic speciation, a phenomenon that is not unknown in the Collembola [41] and not unexpected in Arctic areas as high levels of intra-specific genetic variation have been found in widespread groups genera as *Folsomia* [90]. Furthermore, species-specific and individual responses to climate fluctuations, as well as differences in environmental, climatic conditions, geographical extension and duration of the occupation of the different glacial refugia, would determine post- glacial characteristics of the species and communities in the area [91] making some refugia more readily identifiable than others. Cryptic glacial refugia can nonetheless be detected applying the appropriate method, as repeatedly shown by phylogeographical studies [22,23,41].

Exclusive clusters could be an indicator of either glacial refugia or early post-glacial recolonization, although the high number of species restricted to Beringia would support the glacial refugium hypothesis in line with the accumulating additional evidence of the Beringian glacial refugia [83,86,87]. Abbott *et al.* [83] used haplotype diversity analysis to define Beringia as the major refugium for *Saxifraga oppositifolia* during the LGM, although the high haplotype diversity in Taimyr area was also suggested as being the result of dispersal to the area from different sources rather than resulting from differentiation in glacial refugia. Later studies on the colonization patterns of high Arctic plants [7] have pointed to drifting ice and wood as important high Arctic dispersal vectors, creating patterns such as the link between mid-Siberia and the Svalbard Archipelago, the latter mainly colonized from a refugium east of the Ural Mountains [6]. This agrees with the link shown in Figure 2 between Atlantic and mid-Siberia through cluster 8. The exclusive cluster from the west and mid-Siberian area (D) might therefore reflect the invertebrate community of the glacial refugium described for Arctic plants [6], a hypothesis strengthened by the existence of endemic springtail species in mid-Siberia (D) (Table 3).

## Figures and Tables

**Table 1:** Number of species described from each area and the number of species showing each main type of distribution. Dubious records, as defined in the text, are not included.

		**Area**															
**Number of species**		**A**	**B**	**C**	**D**	**E**	**F**	**G**	**H**	**I**	**a**	**b**	**c**	**d**	**e**	**f**	**H**
**Distribution**	**Total**	159	88	26	150	88	125	126	88	83	51	11	43	30	37	53	45
	**Cosmopolitan**	20	12	3	11	8	13	12	9	15	8	1	4	4	5	6	7
	**Holarctic**	46	34	7	42	24	35	42	31	35	31	7	18	11	13	20	28
	**Palaearctic**	73	37	13	76	36	41	1	0	11	7	2	16	12	12	14	0
	**Nearctic**	1	0	0	0	1	3	35	32	2	0	0	0	0	0	3	7
	**Beringian**	1	4	0	21	19	33	35	15	2	1	1	5	3	7	10	3
	**Atlantic**	18	1	3	0	0	0	1	1	18	4	0	0	0	0	0	0
Exclusive from the area*		52	2	0	18	4	9	22	13	5	0	0	0	0	0	0	0
**Distribution**	**Cosmopolitan**	4	0	-	0	0	0	0	0	1	-	-	-	-	-	-	-
	**Holarctic**	4	0	-	1	0	0	1	0	1	-	-	-	-	-	-	-
	**Palaearctic**	43	2	-	16	4	7	0	0	1	-	-	-	-	-	-	-
	**Nearctic**	0	0	-	0	0	0	18	13	0	-	-	-	-	-	-	-
	**Beringian**	0	0	-	1	0	2	3	0	0	-	-	-	-	-	-	-
	**Atlantic**	1	0	-	0	0	0	0	0	2	-	-	-	-	-	-	-

* Absent from other Arctic areas.

**Table 2:** Number of species per cluster showing different general distribution patterns.

		**Cluster**								
**Number of species**		**1**	**2**	**3**	**4**	**5**	**6**	**7**	**8**	**9**
	**In the cluster**	87	22	19	13	13	11	10	7	7
	**Cosmopolitan**	8	0	0	0	0	0	0	0	1
	**Holarctic**	9	1	1	0	0	0	1	1	6
	**Palaearctic**	54	0	17	11	0	11	0	6	0
	**Nearctic**	0	18	0	0	13	0	9	0	0
	**Beringian**	0	3	1	1	0	0	0	0	0
	**Atlantic**	16	0	0	0	0	0	0	0	0

**Table 3:** List of restricted or endemic species and the area where they occur (*described as Palaearctic but has only been described from the Yenisey river basin).

**Species**	**Area**	
*Folsomia cryptophila* Potapov and Babenko, 2000	D	
*Willemia fjellbergi* Potapov, 1994	D	
*Anurida dynkendga* Babenko, 1998	D	
*Anurida zenokhae* Babenko, 1998*	D	
*Sminthurus sylvestris* Banks, 1899	D	
*Lepidocyrtus tundriensis* Tshelnokov & Bondarenko, 1978	F	
*Anurida parapapillosa* Tshelnokov, 1998	F	
*Sminthurinus henshawi* (Folsom, 1896)	F	
*Micranurida valiana* Fjellberg, 1985	G	
*Protaphorura churchulliana* (Hammer, 1953)	H	
*Proisotoma roberti* Fjellberg, 1991	I	

**Table 4:** Dunn's test pairwise comparisons between areas. Upper right table: pairwise comparisons based on species distribution; Lower left table: pairwise comparisons based on daily temperature recorded between 1996–2005. X: the areas are significantly different (*p* < 0.05). O: differences are not significant (*p* > 0.05).

**Pairwise Comparisons on Presence/Absence of Collembola Species between Areas**								
**Areas**	**A**	**B**	**D**	**E**	**F**	**G**	**H**	**I**
**A**		x	o	x	o	o	x	x
**B**	x		x	o	o	o	o	o
**D**	x	x		x	o	o	x	x
**E**	x	x	o		o	o	o	o
**F**	x	x	o	o		o	o	o
**G**	x	x	x	x	x		o	o
**H**	x	x	x	o	x	x		o
**I**	x	x	x	x	x	x	x	

Pairwise comparisons on monthly aver. temperature measured for the period 1996–2005 between areas.

**Table:** 

0	2																
**Species**		**A**	**B**	**D**	**E**	**F**	**G**	**H**	**I**	**a**	**b**	**c**	**d**	**e**	**f**	**h**	**Cluster**
Hymenaphorura nearctica		0	0	0	1	1	1	0	0	0	0	0	0	0	0	0	N
Hymenaphorura palaearctica Pomorski, 2001		0	0	0	1	1	0	0	0	0	0	0	0	0	1	0	N
Hymenaphorura polonica Pomorski 1990		1	0	0	0	0	0	0	0	0	0	0	0	0	0	0	1
Hymenaphorura sensitiva Pomorski 2001		0	0	0	0	0	1	0	0	0	0	0	0	0	0	0	2
Hymenaphorura sibirica (Tullberg, 1876)		0	1	1	0	0	0	0	0	0	0	0	0	0	0	0	N
Hymenaphorura similis (Folsom, 1917)		0	0	0	1	1	0	0	0	0	0	0	0	0	1	0	N
Hypogastrura actandria Fjellberg, 1988		1	0	0	0	1	1	1	0	0	0	0	0	0	0	1	N
Hypogastrura concolor (Carpenter, 1900)		0	1	1	1	1	1	1	1	1	1	1	1	1	1	1	N
Hypogastrura fjellbergi Babenko & Bulavintsev, 1993		0	0	1	1	1	1	0	0	0	0	1	1	0	0	0	N
Hypogastrura helena Christiansen & Bellinger, 1980		0	0	0	0	0	1	0	0	0	0	0	0	0	0	0	2
Hypogastrura lapponica (Axelson, 1902)		1	0	1	0	1	0	0	0	0	0	0	0	0	0	0	N
Hypogastrura macrotuberculata (Hammer, 1953)		0	0	0	0	0	1	1	0	0	0	0	0	0	0	0	7
Hypogastrura nivicola (Fitch, 1846)		0	0	0	0	0	1	1	0	0	0	0	0	0	0	0	7
Hypogastrura oregonensis Yosii, 1960		0	0	0	0	1	1	1	0	0	0	0	0	0	0	0	N
Hypogastrura perplexa Christiansen & Bellinger, 1980		0	0	0	0	0	1	1	0	0	0	0	0	0	0	0	7
Hypogastrura purpurescens (Lubbock, 1867)|		1	0	0	0	0	0	0	1	0	0	0	0	0	0	0	1
Hypogastrura rangkuli Martynova, 1975		0	0	1	1	0	1	0	0	0	0	0	0	0	0	0	N
Hypogastrura sahlbergi (Reuter, 1895)		1	0	0	0	0	0	0	0	0	0	0	0	0	0	0	1
Hypogastrura sensilis (Folsom 1919)		0	0	1	1	1	1	1	0	1	1	0	1	1	1	1	N
Hypogastrura spei Babenki 1994		0	0	1	0	1	0	0	0	0	0	0	0	0	0	0	6
Hypogastrura tooliki Fjellberg, 1985		0	0	0	0	0	1	0	0	0	0	0	0	0	0	0	2
Hypogastrura trybomi (Schött, 1893)		0	0	1	1	0	0	0	0	0	1	1	1	1	0	0	N
Hypogastrura vernalis (Carl, 1901)		1	0	0	0	0	0	0	0	0	0	0	0	0	0	0	1
Hypohastrura socialis (Uzel, 1891)		1	0	0	0	0	0	0	0	0	0	0	0	0	0	0	1
Isotoma anglicana Lubbock, 1862		1	1	0	0	1	0	0	1	1	0	1	0	0	0	0	N
Isotoma arctica Schött, 1893		0	0	0	0	1	1	0	0	0	0	0	0	0	0	0	N
Isotoma caerulea Bourlet, 1839		0	0	0	0	0	1	0	1	0	0	0	0	0	0	0	N
Isotoma gorodkovi Martynova, 1970		0	0	1	1	1	0	0	0	0	0	0	0	0	1	0	6
Isotoma riparia (Nicolet, 1842)		0	1	1	1	1	0	0	0	0	0	1	0	0	0	0	N
Isotomiella minor (Schäffer, 1896)		1	1	0	0	0	0	1	1	1	0	0	0	0	0	0	N
Isotomodella pusilla Martynova, 1967		1	0	0	0	0	0	0	0	0	0	0	0	0	0	0	1
Isotomodes bisetosus Cassagnau, 1959		1	0	0	0	0	0	0	0	0	0	0	0	0	0	0	1
Isotomurus balteatus (Reuter, 1876)		1	0	0	0	0	0	0	0	0	0	0	0	0	0	0	1
Isotomurus plumosus Bagnall, 1940		0	1	1	0	1	0	0	0	0	0	0	1	0	0	0	N
Kalaphorura bermani (Martynova, 1976)		0	0	0	0	1	1	0	0	0	0	0	0	0	0	0	N
Karlstejnia norvegica Fjellberg, 1974		1	0	0	0	0	0	0	1	0	0	0	0	0	0	0	1
Lepidocyrtus tundriensis Tshelnokov & Bondarenko, 1978		0	0	0	0	1	0	0	0	0	0	0	0	0	0	0	4
Mackenziella psocoides		1	0	0	0	0	0	1	0	0	0	0	0	0	0	0	N
Marisotoma tenuicornis (Axelson, 1903)		1	0	0	0	0	0	0	0	0	0	0	0	0	0	0	1
Megalothorax minimus (Willem 1900)		1	1	1	1	1	1	1	1	1	0	0	1	1	1	1	9
Megaphorura arctica (Tullberg, 1876)		1	1	0	0	0	0	0	1	1	0	1	0	0	0	0	1
Mesaphorura arbeai Simón, Ruiz, Martin et Luciáñez, 1994		0	0	0	0	0	0	0	1	0	0	0	0	0	0	0	N
Mesaphorura critica Ellis, 1976		1	0	0	0	0	0	0	1	0	0	0	0	0	0	0	1
Mesaphorura hylophila Rusek, 1982		1	0	0	0	0	0	0	1	0	0	0	0	0	0	0	1
Mesaphorura italica (Rusek, 1971)		1	0	0	0	0	0	0	1	0	0	0	0	0	0	0	1
Mesaphorura jarmilae Rusek, 1982		1	0	0	0	0	0	0	0	0	0	0	0	0	0	0	1
Mesaphorura jirii Rusek 1982		1	1	0	0	0	0	0	1	1	0	0	0	0	0	0	1
Mesaphorura krausbaueri Börner, 1901		1	0	0	0	0	0	0	1	0	0	0	0	0	0	0	1
Mesaphorura macrochaeta Rusek, 1976		1	1	0	1	1	0	0	1	1	0	0	0	0	1	1	N
Mesaphorura petterdassi (Fjellberg, 1988)		1	0	0	0	0	0	0	1	0	0	0	0	0	0	0	1
Mesaphorura sylvatica (Rusek, 1971)		1	0	0	0	0	0	0	0	0	0	0	0	0	0	0	1
Mesaphorura tenuisensillata Rusek, 1974		1	1	1	0	0	0	0	1	1	0	0	0	0	0	0	N
Mesaphorura yosii (Rusek, 1967)		1	0	0	0	0	0	0	0	0	0	0	0	0	0	0	1
Metaphorura affinis (Börner, 1902)		1	0	0	0	0	0	0	0	0	0	0	0	0	0	0	1
Metisotoma grandiceps (Reuter, 1891)		0	0	1	0	1	1	1	0	0	0	0	0	0	1	0	N
Micranurida forsslundi Gisin, 1949		1	0	0	0	0	0	0	0	0	0	0	0	0	0	0	1
Micranurida pygmaea Börner, 1901		1	1	1	0	1	1	1	1	1	0	0	0	0	0	1	N
Micranurida spirillifera Hammer, 1953		0	0	0	0	0	1	1	0	0	0	0	0	0	0	0	7
Micranurida valiana Fjellberg, 1985		0	0	0	0	0	1	0	0	0	0	0	0	0	0	0	2
Micranurophorus musci Bernard, 1977		1	0	0	0	0	0	0	0	0	0	0	0	0	0	0	1
Micraphorura absoloni (Börner, 1901)		1	1	0	0	1	1	1	1	0	0	0	0	0	0	0	N
Micraphorura alnus (Fjellberg, 1987)		0	0	0	0	1	0	0	0	0	0	0	0	0	1	0	N
Micraphorura interrupta (Fjellberg, 1987)		0	0	0	1	0	0	0	0	0	0	0	0	0	0	0	N
Micraphorura nataliae (Fjellberg, 1987)		0	0	0	0	1	0	0	0	0	0	0	0	1	0	0	N
Mitchellania gibbomucronata (Hammer, 1953)		0	0	0	0	0	0	1	0	0	0	0	0	0	0	0	5
Mitchellania horrida (Yossi, 1960)		0	0	0	0	0	1	0	0	0	0	0	0	0	0	0	2
Mitchellaria loricata (Yosii, 1960)		0	0	0	0	0	1	0	0	0	0	0	0	0	0	0	2
Morulina mackenziana Hammer, 1953		0	0	0	0	0	1	1	0	0	0	0	0	0	0	1	N
Morulina theeli Babenko & fjellberg, 2001		0	0	1	0	0	0	0	0	0	0	0	0	0	0	0	3
Morulina thulensis Hammer, 1953		0	0	0	0	1	1	1	0	0	0	0	0	0	1	0	N
Morulodes serratus (Folsom, 1916)		0	0	0	0	0	1	0	0	0	0	0	0	0	0	0	2
Mucrella denali (Fjellberg, 1985)		0	0	0	0	1	1	0	0	0	0	0	0	0	0	0	N
Mucrella navicularis (Schött, 1893)		0	0	1	1	0	0	0	0	0	0	0	0	0	0	0	N
Multivesicula dolomitica Rusek, 1982		0	0	1	0	1	0	1	0	0	0	0	0	0	1	0	N
Oligaphorura groenlandica (Tullberg, 1876)		1	1	1	1	1	1	1	1	1	1	1	1	1	1	1	9
Oligaphorura nuda (Fjellberg, 1987)		0	0	0	1	0	1	0	0	0	0	0	0	0	0	0	N
Oligaphorura pingicola (Fjellberg, 1987)		0	0	0	0	0	1	0	0	0	0	0	0	0	0	0	2
Oligaphorura reversa (Fjellberg, 1987)		0	0	0	0	0	1	0	0	0	0	0	0	0	0	0	2
Oligaphorura schoetti (Lie- Pettersen, 1896)		1	0	0	0	0	0	0	0	0	0	0	0	0	0	0	1
Oligaphorura ursi (Fjellberg, 1984)		1	1	1	0	1	1	0	1	1	0	1	0	0	0	0	N
Pachyotoma crassicauda (Tullberg, 1871)		0	1	1	0	0	1	0	0	0	0	1	0	0	0	0	N
Paranura quadrilobata Hammer, 1953		0	0	0	0	1	1	1	0	0	0	0	0	0	0	0	N
Paranura sexpunctata Axelson, 1902		1	0	0	0	0	0	0	0	0	0	0	0	0	0	0	1
Parisotoma agrelli (Delamare Debuotteville, 1950)		1	0	0	0	0	0	0	0	0	0	0	0	0	0	0	1
Parisotoma ekmani (Fjellberg, 1977)		1	1	1	0	1	1	1	1	0	0	0	0	0	0	1	N
Parisotoma longa (Potapov, 1991)		0	0	1	0	1	0	0	0	0	0	0	0	0	0	0	6
Parisotoma notabilis (Schäffer, 1896)		1	1	1	0	1	1	1	1	1	0	1	0	0	0	0	N
Parisotoma reducta (Rusek, 1984)		0	1	1	1	1	0	0	0	0	0	0	0	1	0	0	N
Parisotoma trichaetosa (Martynova, 1977)		0	0	0	0	1	0	0	0	0	0	0	0	0	0	0	4
Podura aquatica Linnaeus, 1758		1	1	1	1	1	1	1	0	0	0	0	1	1	1	1	N
Pogonognathellus lividus (Tullberg, 1876)		0	0	1	0	1	0	0	0	0	0	0	0	0	0	0	6
Proisotoma ananevae Babenko & Bulavintsev, 1993		0	0	1	0	1	0	0	0	0	0	1	0	0	1	0	N
Proisotoma buddenbrocki strenzke, 1954		1	0	0	0	0	0	0	0	0	0	0	0	0	0	0	1
Proisotoma clavipila (Axelson, 1903)		1	0	0	0	0	0	0	0	0	0	0	0	0	0	0	1
Proisotoma ladaki Denis, 1936		0	0	1	0	0	0	0	0	0	0	0	0	0	0	0	3
Proisotoma minima (Absolon, 1901)		1	0	0	0	0	0	0	0	0	0	0	0	0	0	0	1
Proisotoma minuta (Tullberg, 1871)		1	0	0	0	0	0	0	0	0	0	0	0	0	0	0	1
Proisotoma roberti		0	0	0	0	0	0	0	1	0	0	0	0	0	0	0	N
Proisotoma subarctica Gisin, 1950		1	0	1	0	0	0	0	0	0	0	1	0	0	0	0	N
Protaphorura armata (Tullberg, 1986)		1	0	0	0	0	0	0	0	0	0	0	0	0	0	0	1
Protaphorura bicampata (Gisin, 1956)		1	0	1	0	0	0	0	0	0	0	0	0	0	0	0	N
Protaphorura boedvarssoni Pomorski, 1993		1	1	1	0	0	0	0	0	0	0	0	0	0	0	0	8
Protaphorura borealis (Martynova, 1973)		0	0	1	1	1	1	0	0	0	0	0	0	0	1	0	N
Protaphorura campata		0	0	0	0	0	0	0	1	0	0	0	0	0	0	0	N
Protaphorura cancellata (Gisin, 1956)		1	0	1	0	0	0	0	0	0	0	1	1	0	0	0	N
Protaphorura churchulliana (Hammer, 1953)		0	0	0	0	0	0	1	0	0	0	0	0	0	0	0	5
Protaphorura duodecimopunctata (Folsom, 1919)		0	0	0	0	0	1	1	0	0	0	0	0	0	0	0	7
Protaphorura islandica (Bödvarsson, 1959)		1	0	0	0	0	0	0	0	0	0	0	0	0	0	0	1
Protaphorura jacutica (Martynova, 1976		0	1	1	1	0	0	0	0	0	0	0	0	0	0	0	N
Protaphorura macfadyeni (Gisin, 1953)		1	0	0	0	0	0	0	1	1	0	0	0	0	0	0	1
Protaphorura madrodentata (Hammer, 1953)		0	0	0	0	0	0	1	0	0	0	0	0	0	0	1	N
Protaphorura neriensis (Martynova, 1976)		0	0	0	1	1	0	0	0	0	0	0	0	0	0	0	4
Protaphorura paucisetosa (Hammer, 1953)		0	0	0	0	0	0	1	0	0	0	0	0	0	0	0	5
Protaphorura pjasinae (Martynova, 1976)		0	0	1	0	1	0	0	0	0	0	1	1	1	1	0	N
Protaphorura procampata (Gisin, 1956)		1	1	0	0	0	0	0	1	0	0	0	0	0	0	0	1
Protaphorura pseudoarmata (Folsom, 1917)		0	0	0	0	0	1	1	1	0	0	0	0	0	0	0	N
Protaphorura pseudovanderdrifti (Gisin, 1957)		1	0	0	0	0	0	0	1	0	0	0	0	0	0	0	1
Protaphorura pulvinata (Gisin, 1954)		0	1	1	0	0	0	0	0	0	0	0	0	0	0	0	N
Protaphorura subartica (Martynova, 1976)		0	1	1	1	0	0	0	0	0	0	1	0	1	0	0	N
Protaphorura subuliginata Gisin, 1956		1	1	0	0	0	0	0	1	0	0	0	0	0	0	0	1
Protaphorura taimiryca (Martynova, 1976)		0	0	1	0	0	0	0	0	0	0	1	0	0	0	0	N
Protaphorura tricampata (Gisin, 1956)		1	0	0	0	0	0	0	0	0	0	0	0	0	0	0	1
Protaphorura tschernovi (Martynova, 1976)		0	0	1	0	0	0	0	0	0	0	0	0	0	0	0	3
Protaphorura tundricola (Martynova, 1976)		0	0	1	0	0	0	0	0	0	0	0	0	0	0	0	3
Protaphorura vanderdrifti (Gisin, 1952)		0	1	0	0	0	0	0	0	0	0	0	0	0	0	0	N
Pseudachorutella asigillata (Börner, 1901)		1	1	0	0	0	0	0	0	0	0	0	0	0	0	0	1
Pseudachorutes corticicolus (Schäffer, 1896)		1	0	0	0	0	0	0	0	0	0	0	0	0	0	0	1
Pseudachorutes dubius Krausbauer, 1898		1	1	0	0	0	0	0	0	0	0	0	0	0	0	0	1
Pseudachorutes sibiricus Rusek, 1991		0	1	1	1	1	1	0	0	0	0	0	0	0	0	0	N
Pseudachorutes subcrassus Tullberg, 1871		1	0	0	0	0	0	0	0	0	0	0	0	0	0	0	1
Pseudanurophorus alticolus Bagnall, 1949		1	0	1	0	1	0	0	1	1	0	0	1	0	0	0	N
Pseudanurophorus arcticus Christiansen, 1951		0	0	1	0	1	1	1	0	0	0	0	0	0	0	0	N
Pseudanurophorus binoculatus Kseneman 1934		1	1	1	0	1	1	0	1	1	0	0	0	0	0	1	N
Pseudoisotoma sensibilis (Tullberg, 1876)		1	1	1	1	1	1	1	1	1	0	1	0	0	1	1	9
Pseudonychiurus dentatus (Folsom, 1902)		0	0	0	0	0	1	0	0	0	0	0	0	0	0	0	2
Psyllaphorura sensillifera (Martynova, 1981)		0	0	0	1	1	0	0	0	0	0	0	0	0	0	0	4
Psyllaphorura uenoi (Yosii, 1954)		0	0	0	0	0	1	0	0	0	0	0	0	0	0	0	2
Ptenothrix palmatus (Folsom, 1902)		0	0	0	0	0	1	0	0	0	0	0	0	0	0	0	2
Schaefferia oculea Babenko, 1999		0	0	0	1	0	0	0	0	0	0	0	0	0	0	0	N
Schoettella ununguiculata (Tullberg, 1869)		0	0	0	0	0	0	1	1	0	0	0	0	0	0	0	N
Secotomodes sibiricus Potapov, 1988		0	0	1	0	0	0	0	0	0	0	0	0	0	0	0	3
Sminthurides malmgreni (Tullberg, 1876)		1	1	1	1	1	1	1	1	1	0	1	0	1	1	1	9
Sminthurides occultus Mills, 1934		0	0	0	0	0	0	1	0	0	0	0	0	0	0	0	5
Sminthurides parvulus (Krausbauer, 1898)		1	1	1	1	1	0	0	0	0	0	0	0	0	0	0	N
Sminthurides pseudassimilis Stach, 1956		1	0	0	0	0	0	0	0	0	0	0	0	0	0	0	1
Sminthurides schoetti Axelson, 1903		1	0	1	1	1	0	0	1	0	0	1	0	0	1	0	N
Sminthurinus albifrons (Tullberg, 1871)		1	0	0	0	0	0	0	0	0	0	0	0	0	0	0	1
Sminthurinus bimaculatus Axelson, 1902		1	1	0	0	0	0	0	0	0	0	0	0	0	0	0	1
Sminthurinus elegans (Fitch, 1863)		1	0	0	0	0	1	0	0	0	0	0	0	0	0	0	N
Sminthurinus henshawi (Folsom, 1896)		0	0	0	0	1	0	0	0	0	0	0	0	0	0	0	4
Sminthurinus quadrimaculatus (Ryder, 1879)		0	0	0	0	0	1	1	0	0	0	0	0	0	0	0	7
Sminthurus incisus Snider, 1978		0	0	0	0	0	1	0	0	0	0	0	0	0	0	0	2
Sminthurus multipunctatus Schäffer, 1896		0	0	1	1	0	0	0	0	0	0	0	0	0	0	0	N
Sminthurus nigromaculatus Tullberg, 1871		0	0	1	0	0	0	0	1	0	0	0	0	0	0	0	N
Sminthurus orientalis Bretfeld, 2000		0	0	0	1	1	0	0	0	0	0	0	0	0	1	0	N
Sminthurus sylvestris Banks, 1899		0	0	1	0	0	0	0	0	0	0	0	0	0	0	0	3
Sminthurus variegatus Tullberg, 1876		0	0	1	0	1	0	0	0	0	0	0	0	0	0	0	6
Sphaeridia leutrensis Dunger & Bretfeld, 1989		0	0	1	0	0	0	0	0	0	0	0	0	0	0	0	3
Sphaeridia pumilis (Krausbauer, 1898)		1	1	1	1	1	1	1	1	1	0	0	0	0	0	1	N
Stachanorema tolerans Babenko, 1994		0	0	0	0	0	0	1	0	0	0	0	0	0	0	1	N
Stenacidia violacea (Reuter, 1881)		1	0	1	0	1	0	0	0	0	0	0	1	0	1	0	N
Stenaphorurella quadrispina (Börner, 1901)		0	1	0	0	0	0	0	0	0	0	0	0	0	0	0	N
Supraphorura furcifera (Börner, 1901)		0	1	1	0	0	0	0	0	0	0	1	0	0	0	0	N
Tantulonychiurus volinensis (Szeptycki, 1964)		0	0	1	1	1	0	0	0	0	0	0	0	0	0	0	6
Tetracanthella arctica Cassagnau 1959		1	0	0	0	0	0	0	0	1	0	0	0	0	0	1	N
Tetracanthella martynovae Potapov, 1997		0	0	0	1	1	0	0	0	0	0	0	0	0	0	0	4
Tetracanthella orientalis Martynova, 1977		0	0	0	0	1	0	0	0	0	0	0	0	0	0	0	4
Tetracanthella pilosa Schött, 1891		1	0	0	0	0	0	0	0	0	0	0	0	0	0	0	1
Tetracanthella sibirica Deharveng, 1987		0	0	0	1	1	1	1	1	0	0	0	0	1	1	0	N
Tetracanthella wahlgreni Axelson, 1907		1	1	1	0	0	0	0	0	0	0	1	0	0	0	0	8
Thalassaphorura debilis (Moniez, 1890)		1	0	0	0	0	1	0	1	0	0	0	0	1	0	1	N
Thalassaphorura duplopunctata (Strenzke, 1954)		1	0	0	0	0	1	0	1	0	0	0	0	1	0	1	N
Tomocerus minutus Tullberg, 1876		1	1	1	0	1	1	0	0	0	0	0	0	0	0	0	N
Tomocerus sibiricus Reuter, 1891		0	1	1	0	0	0	0	0	0	0	0	0	0	0	0	N
Tomocerus vulgaris (Tullberg, 1871)		0	0	1	0	0	0	0	0	0	0	0	0	0	0	0	3
Uralaphorura schilovi (Martynova, 1976)		1	0	1	1	0	0	0	0	0	0	1	0	0	0	0	N
Uzellia hansoni Mills & Richards, 1953		0	0	0	0	0	1	1	0	0	0	0	0	0	0	0	7
Vertagopus arcticus Martynova, 1969		1	0	1	1	1	0	0	1	1	0	0	1	1	1	0	N
Vertagopus brevicaudatus (Carpenter, 1900)		0	0	1	0	0	0	0	0	0	1	0	1	0	0	1	N
Vertagopus cinereus (Nicolet, 1842)		1	0	0	0	0	0	0	1	0	0	0	0	0	0	0	1
Vertagopus reuteri (Schött, 1893)		0	0	0	0	1	0	0	0	0	0	0	0	0	0	0	4
Vertagopus Sarekensis (Whalgren, 1906)		1	0	0	0	0	0	0	0	0	0	0	0	0	0	0	1
Vertagopus westerlundi (Reuter, 1897)		1	0	0	0	0	0	0	0	0	0	0	0	0	0	0	1
Wankeliella intermedia Potapov & Stebaeva, 1997		0	0	1	0	0	0	0	0	1	0	0	0	0	0	0	N
Wankeliella medialis Simón & Jordana, 1994		1	0	0	0	0	0	0	0	0	0	0	0	0	0	0	1
Weberacantha octa Christiansen, 1951		0	0	0	0	1	1	1	1	0	0	0	0	0	0	0	N
Willemia anophthalma Börner 1901		1	0	1	1	1	1	1	1	1	0	0	0	0	0	1	N
Willemia arida Fjellberg, 1991		0	0	0	0	0	1	0	0	0	0	0	0	0	0	0	2
Willemia denisi Mills, 1932		1	1	1	0	1	1	1	1	0	0	0	0	0	1	0	N
Willemia fjellbergi Potapov, 1994		0	0	1	0	0	0	0	0	0	0	0	0	0	0	0	3
Willemia multilobata Gers & Deharveng, 1985		0	0	1	0	0	0	0	0	0	0	0	0	0	0	1	N
Willemia scandinavica Stach, 1949		1	0	1	0	0	1	1	1	1	1	0	1	0	1	1	N
Willemia similis Mills, 1934		0	1	1	1	1	1	1	1	1	0	0	1	1	1	1	N
Willowsia nigromaculata (Lubbock, 1873)		1	0	1	0	1	0	1	0	0	0	0	0	0	0	0	N
Xenylla boernery Axelson, 1903		1	0	0	0	0	0	0	0	0	0	0	0	0	0	0	1
Xenylla brevicaudata Tullberg, 1869		1	1	0	0	0	0	0	0	0	0	0	0	0	0	0	1
Xenylla canadensis Hammer, 1953		0	0	0	0	0	0	1	0	0	0	0	0	0	0	0	5
Xenylla corticales Börner 1901		1	0	0	0	0	0	0	0	0	0	0	0	0	0	0	1
Xenylla grisea Axelson, 1900		1	0	0	0	0	0	0	1	0	0	0	0	0	0	0	1
Xenylla humicola (Fabricius, 1780)		1	1	1	0	1	0	1	1	1	0	1	0	0	0	0	N
Xenylla maritima Tullberg 1869		1	1	0	0	0	0	0	0	0	0	0	0	0	0	0	1
Xenylla martynovae Dunger, 1983		0	0	1	0	0	0	0	0	0	0	0	0	0	0	0	3
Xenylla xavieri Gama, 1959		1	0	0	0	0	0	0	0	0	0	0	0	0	0	0	1
Xenyllodes armatus Axelson 1903		1	1	1	1	1	1	1	0	1	0	1	0	0	0	0	N
Xenyllodes wapti fjellberg, 1985		0	0	0	0	0	1	0	0	0	0	0	0	0	0	0	2
